# Dr. J. D. Skeer’s Adhesive Splints

**Published:** 1878-07

**Authors:** A. H. Foster


*Dr. J. D. Skeer's Adhesive Splints.*

A strip of adhesive plaster, one half to one inch wide, is laid lengthwise, face side up, on the face of the splint (which has been previously covered with muslin), and fastened firmly by stitches through each end.

In applying this splint to the surface of the limb, the plaster, by adhering, will retain it in place, and prevent its slipping laterally or lengthwise.

In this way splints can be retained in position, where they could not well be by any method of adhesive strips placed across the back of the splints. They are especially applicable to fractures of the thigh in infants and children, being retained in position by the "many tailed bandage" tied over the first splint. In this way Dr. Skeer has used them for the past five or six years with entire satisfaction.

Dr. Skeer does not claim absolute priority in this, but, so far as he is aware, it is original with him.

The writer, from his own experience, can testify to its value in such cases. A. H. Foster, M. D.

